# Pharmacotechnical, Physico-Chemical, and Antioxidant Evaluation of Newly Developed Capsule Formulations

**DOI:** 10.3390/ijms241411426

**Published:** 2023-07-13

**Authors:** Emma Adriana Ozon, Izabela Dana Maria Iuga, Magdalena Mititelu, Adina Magdalena Musuc, Bogdan Nicolae Manolescu, Simona Petrescu, Jeanina Pandele Cusu, Adriana Rusu, Vasile-Adrian Surdu, Eliza Oprea, Sorinel Marius Neacșu, Oana Karampelas, Viviana Elian

**Affiliations:** 1 Department of Pharmaceutical Technology and Biopharmacy, Faculty of Pharmacy, “Carol Davila” University of Medicine and Pharmacy, 6 Traian Vuia Street, 020945 Bucharest, Romania; emma.budura@umfcd.ro (E.A.O.); oana.karampelas@umfcd.ro (O.K.); 2 Department of Clinical Laboratory and Food Safety, Faculty of Pharmacy, “Carol Davila” University of Medicine and Pharmacy, 6 Traian Vuia Street, 020945 Bucharest, Romania; izabela-dana-maria.iuga@mst.umfcd.ro; 3 “Ilie Murgulescu” Institute of Physical Chemistry, 202 Spl. Independentei, 060021 Bucharest, Romania; simon_pet@icf.ro (S.P.); jeanina@icf.ro (J.P.C.); arusu@icf.ro (A.R.); 4 ”C. Nenitescu” Department of Organic Chemistry, Faculty of Applied Chemistry and Science of Materials, University “Politehnica” of Bucharest, 1–7 Polizu Street, 011061 Bucharest, Romania; bogdan.manolescu@upb.ro; 5 Department of Science and Engineering of Oxide Materials and Nanomaterials, Faculty of Chemical Engineering and Biotechnologies, University “Politehnica” of Bucharest, 1–7 Polizu Street, 011061 Bucharest, Romania; adrian.surdu@upb.ro; 6 Department of Microbiology, Faculty of Biology, University of Bucharest, 1–3 Portocalilor Way, 060101 Bucharest, Romania; eliza.oprea@g.unibuc.ro; 7 Professional Farma Line, 116 Republicii Street, 105200 Baicoi, Romania; neacsusorinelmarius@gmail.com; 8 Department of Diabetes, Nutrition and Metabolic Diseases “Carol Davila” University of Medicine and Pharmacy, INDNBM N.C. Paulescu, 5–7 Ion Movila Street, 030167 Bucharest, Romania; viviana.elian@umfcd.ro

**Keywords:** melatonin, coenzyme Q10, resveratrol, quercetin, biotin, antioxidant supplements

## Abstract

The excess of free radicals causes numerous imbalances in the body that lead to premature aging, the degradation of internal structures, and the appearance of numerous pathologies responsible for the increased risk of premature death. The present work aims to evaluate the physical, chemical, pharmacotechnical, and antioxidant activity of newly achieved capsule formulations. These two formulations were *F1_a.i_*_._, which contains melatonin:biotin:coenzyme Q10 (weight ratio of 1:2:60), and *F2_a.i._,* which contains quercetin:resveratrol:biotin:coenzyme Q10 (weight ratio of 10:10:1:10). The adequate selection of the excipient types and amounts for final capsule formulations (*F1_c.c_*_._, *F2_c.c_*_._) was based on preformulation studies performed on the powders containing active ingredients. The antioxidant activity assessed using three methods (ABTS, DPPH, and FRAP) compared with acid ascorbic as a positive control demonstrated that the *F2_c.c_*_._ formulation possesses the strongest antioxidant capacity. The results confirmed the suitable formulation and the accurate selection of the types and amounts of active ingredients, as well as the auxiliary excipients used in newly developed capsule formulations as supplements with an excellent antioxidant effect on the human body.

## 1. Introduction

Industrial progress and the increase in digitalization of various activity sectors have led to the acceleration of pollution worldwide and the appearance of additional sources of free radicals. In the context of certain problems related to the nutritional degradation of the soil with negative effects on the nutritional value of vegetables and fruits, there is more and more talk about supplementing food with effective sources of antioxidants [1,2].

The human body produces a series of natural enzymatic antioxidants with a role in neutralizing free radicals such as superoxide dismutase, glutathione peroxidase, catalase, and methionine reductase, but often these are not sufficient to protect cells from the negative effects of oxidative stress such as premature aging and the occurrence of cardiovascular and degenerative diseases [3,4]. Excessive pollution, radiation, cigarette smoke, processed foods, alcohol consumption, saturated fats, and sweets are the main causes of the excessive generation of free radicals. Oxidative stress occurs as a result of the imbalance between the excessive generation of free radicals and the insufficient production of natural enzymatic antioxidants in the body [5,6]. The excess of free radicals in the body produces lipid peroxidation, the degradation of cell membranes, and the degradation of DNA and of its own proteins, causing serious diseases: Diabetes, atherosclerosis, inflammatory diseases, neurodegenerative diseases (Parkinson, Alzheimer’s), cancer, etc. The most vulnerable organs to the aggression of free radicals are nerve cells, the lens, blood vessels, and the pancreas, which have a reduced capacity to inactivate them [7,8].

To prevent the devastating effects of oxidative stress, daily consumption of fruits and vegetables, whole grains, and spices is recommended, and in special situations (activity in the presence of radiation sources, excessively polluted areas, intense physical activity, and intense intellectual activity), it is recommended to supplement the diet with antioxidant supplements [9,10,11,12]. Vegetables and fruits are also important sources of fiber, along with antioxidants, contributing to the alkalization and detoxification of the body [13]. Other foods rich in antioxidants are seafood and bee honey (especially forest honey and Manuka honey). It is very important to consider the quality of food in addition to choosing cooking methods that protect antioxidants because pollutants such as pesticides, heavy metals, and microplastics are generators of free radicals [14,15].

Clinical studies have highlighted beneficial effects in combating the effects of oxidative stress in the case of diets rich in vegetables and fruits, but also in the case of supplementing diets with antioxidant supplements. Moreover, avoiding sources of oxidants (cigarettes, alcohol, nutrient-poor and excessively processed foods, stress, etc.) should be considered as important as an antioxidant-rich diet [16,17,18,19].

Melatonin is a natural hormone with a role in regulating the circadian rhythm but is also a powerful antioxidant that stimulates the skin’s natural defense system, helping to fight free radicals [20,21]. Coenzyme Q10 is produced naturally by the human body. Being an effective antioxidant, it protects the brain, heart, and muscular system. Practically, it is present in all body cells, with the highest concentration in the mitochondria where it participates in energy production and stimulates biochemical reactions [22,23]. The levels of coenzyme Q10 and melatonin in the body decrease with age, which is why taking them in the form of a food supplement can benefit the body.

Quercetin is a flavonoid found in vegetables and fruits. It has strong antioxidant properties. This is why it is recommended as a food supplement for multiple conditions: neurodegenerative diseases, cardiovascular diseases, prevention of premature aging, oncological diseases, strengthening of the immune system, etc. [24,25].

Resveratrol is a compound belonging to the class of stilbenoids, a polyphenol produced by various plants, especially dark-colored fruits (black grapes, blueberries, blackberries, raspberries, etc.). Over time, clinical studies have demonstrated the powerful antioxidant role of resveratrol and the effects it can produce in the body, from slowing the aging process of the skin to protecting the health of the heart, brain, and cognitive functions. Resveratrol has become increasingly known for its anti-inflammatory and antiaging properties, but also for its contribution to combating several ailments [26,27].

Biotin (vitamin H) is a water-soluble vitamin that belongs to the vitamin B complex and acts as a coenzyme for biotin-dependent carboxylases in humans. The role of biotin in the human body is complex, having stimulatory effects reducing hypertension, hypertriglyceridemia, and hyperglycemia. Biotin stimulates the absorption of glucose in the liver, the secretion of insulin, and the synthesis of glycogen, all of which are determined by the level of glucose [28]. It has been demonstrated that biotin deficiency is correlated with peripheral insulin resistance and hyperglycemia in humans. In contrast, biotin supplementation in pharmacological formulations has proved an increased hypoglycemic and antioxidant role [29].

The novelty of the present research is based on the development of two new formulations in the form of capsules and their physico-chemical and pharmacotechnical properties and the antioxidant activity evaluation. The physical and chemical properties in terms of Fourier Transform Infrared Spectroscopy, X-ray diffraction analysis, thermogravimetry, and scanning electron microscopy were evaluated in order to assess the compatibility between the active ingredients and the excipients. Subsequently, antioxidant activity was evaluated to demonstrate the antioxidant effect of the two pharmaceutical formulations.

## 2. Results

### 2.1. Physical and Chemical Evaluation

#### 2.1.1. FTIR Analysis

**Figure 1:**
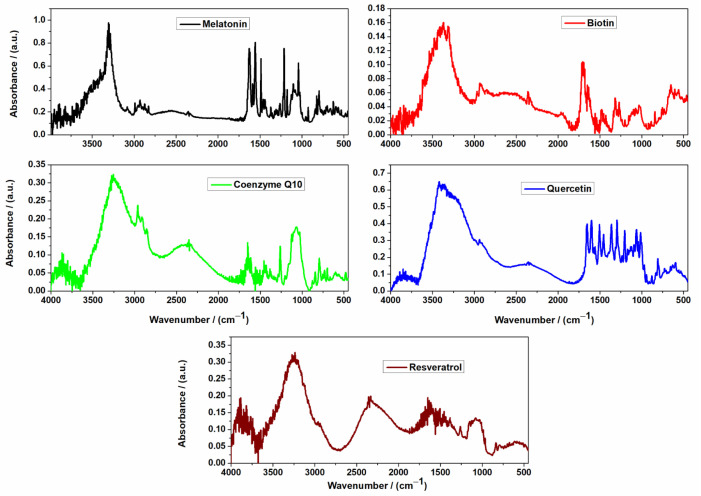
FTIR spectra of active ingredients.

**Figure 2:**
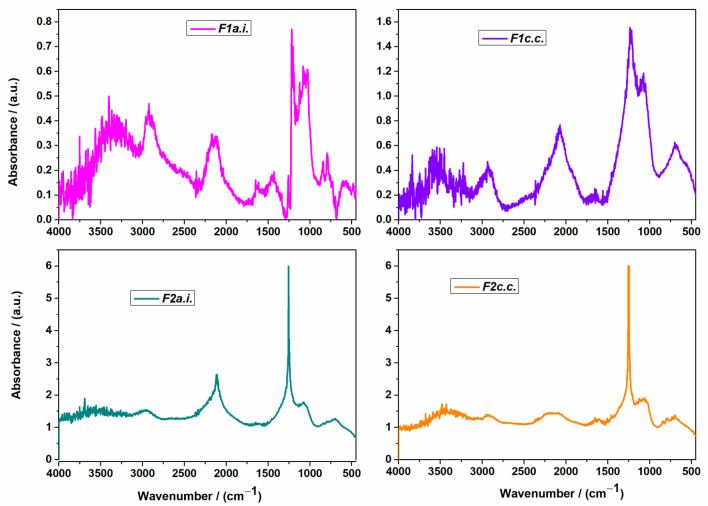
FTIR spectra of capsule formulations *F1a.i.*; *F1c.c.*; *F2a.i*.; *F2c.c*..

The FTIR spectra of the investigated compounds are represented in Figure 1 for the active ingredients, and for the capsule formulations without and with excipients in Figure 2.

The FTIR spectrum of melatonin (black line from Figure 1) revealed the following characteristic peaks: The NH stretching peak at 3300 cm^−1^, the amidic carbonyl group CO stretching observed at 1550 cm^−1^, and the characteristic peaks of melatonin at 1000–1200 cm^−1^ attributed to the C–O stretching [30]. The FTIR spectrum of biotin (red line from Figure 1) showed the following absorption peaks: The O–H stretching vibration bands at 3500–3200 cm^−1^, stretching vibration band of –CH_3_ and –CH_2_ groups around 2925 cm^−1^; the stretching vibration of the carboxyl C = O at approximately 1730 cm^−1^; the stretching vibration of the carbonyl group (amide I peak), N–H bending and tensile vibration (amide II peak), and C–N bending vibration (amide III peak) at 1685, 1485, and 1260 cm^−1^ [31,32]. In the FTIR spectrum of coenzyme Q10, a series of its characteristic peaks were identified (green line from Figure 1): The peaks between 2909 and 2964 cm^−1^ were assigned to the stretching vibration of saturated C–H at 2849 cm^−1^ attributed to the stretching vibration of saturated C–O, at 1609 and 1648 cm^−1^ attributed to the vibration of the typical benzoquinone ring, at 1448 cm^−1^ attributed to the out-of-plane bending vibration of C–H, and between 1153 and 1287 cm^−1^ attributed to the out-of-plane bending vibration of C–O on the benzoquinone ring; the stretching vibration of C–O–C was observed between 1024 and 1104 cm^−1^ and the stretching vibration of C = H was observed between 795 and 877 cm^−1^ [33,34].

The FTIR spectrum of quercetin from Figure 1 (blue line) shows the following characteristic bands: The wide O-H stretching vibration absorption peak of 3446 cm^−1^; OH bending of the phenol function at 1379 cm^−1^; the carbonyl C = O aryl ketonic stretch absorption at 1666 cm^−1^; the C = C aromatic ring stretch bands at 1610, 1560, and 1510 cm^−1^; the in-plane bending band of C–H in aromatic hydrocarbon at 1317 cm^−1^; the out-of-plane bending bands at 933, 820, 679, and 600 cm^−1^; the C–O stretching in the aryl ether ring, the C–O stretching in phenol, and the C–CO–C stretch and bending in ketone at 1263, 1200, and 1165 cm^−1^, respectively [35,36,37].

The FTIR spectrum of resveratrol (wine line from Figure 1) revealed intense absorption bands at 1608 cm^−1^ attributed to the aromatic C-C double-bond stretching, at 1592 cm^−1^ assigned to the olefinic C-C stretching, and at 966 cm^−1^ attributed to the typical trans olefinic band [38].

In Figure 2, the FTIR spectra of capsule formulations with active ingredients and final formulations with excipients are represented.

The FTIR spectra of capsule formulations showed a reduction in the peak intensities and also the disappearance of the characteristic peaks of their active ingredients, which demonstrated the possible interaction that takes place between the compounds. The FTIR spectra of the final capsule formulation are quite similar to the formulations without excipients with only a reduction in the intensities of some peaks. This demonstrates that the excipients have no influence on the structure of the formed compounds. They only act as fillers, superdisintegrants, and lubricants.

#### 2.1.2. XRD Analysis

**Figure 3:**
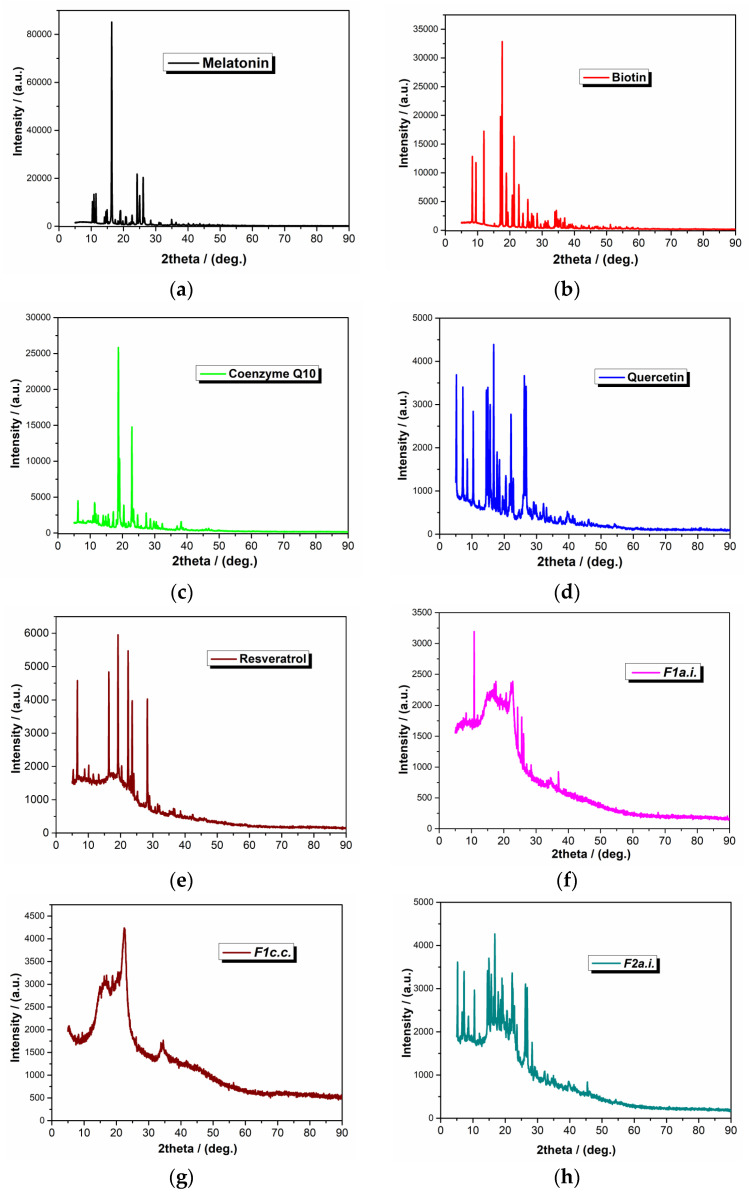

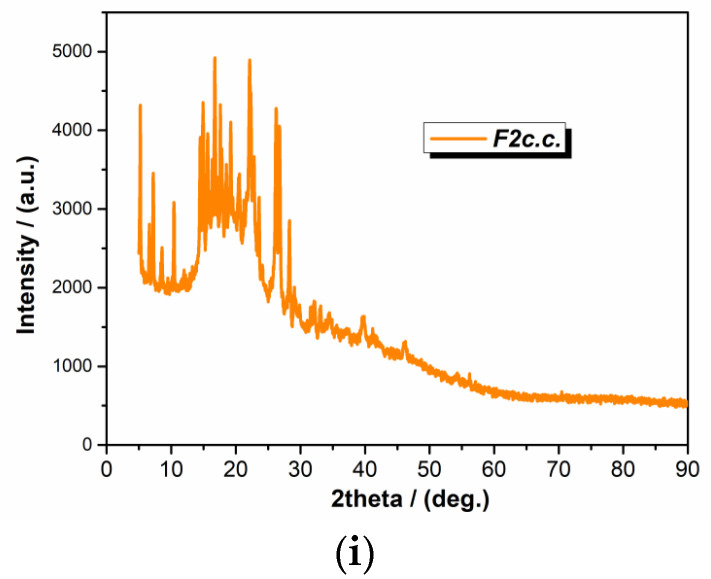
X-ray diffractograms of the compounds (**a**) melatonin; (**b**) biotin; (**c**) coenzyme Q10; (**d**) quercetin; (**e**) resveratrol; (**f**) *F1a.i.*; (**g**) *F1c.c.*; (**h**) *F2a.i.*; (**i**) *F2c.c.*.

X-ray diffraction was further used to investigate the crystalline nature of active ingredients. The X-ray diffraction patterns of active ingredients and capsule formulations are represented in Figure 3. The X-ray diffractogram of melatonin (Figure 3a), biotin (Figure 3b), coenzyme Q10 (Figure 3c), quercetin (Figure 3d), and resveratrol (Figure 3e) presented intense crystalline peaks between 3° and 50° and some other peaks of weak intensity indicating their crystalline form. The XRD spectrum of melatonin (Figure 3a) presents some sharp peaks at the diffraction angles of 2θ = 17.8, 24.8, 25.2, and 26.2 suggesting its crystalline structure [39].

The characteristic peaks of quercetin (Figure 3d) are primarily at 2θ = 6.3, 7.3, 10.8, 12.5, 13.6, 14.3, 15.7, 18.0, 24.4, and 27.3° [40]. XRD pattern of resveratrol revealed (Figure 3e) intense characteristic peaks at diffraction angles 2θ = 6.6, 16.4, 19.1, 22.3, and 28.3° [41].

The typically observed peaks for the active ingredients are in agreement with the literature data, which demonstrate that these compounds had a well-formed crystalline structure and well-organized atomic arrangement.

The XRD diffractograms of the capsule formulations show a reduction in the height and number of the peaks, and also the disappearance of the main characteristic peaks, indicating reduced crystallinity of the capsule. A decrease in capsule formulation crystallinities confirmed the occurrence of some kind of interaction by combining the active ingredients with the selected excipients, as was expected, but which cannot negatively affect the active ingredients. The XRD results confirmed the transformation of crystalline ingredients into their amorphous form of final formulation due to the processing method used for their preparation.

#### 2.1.3. Thermogravimetric Analysis

**Figure 4:**
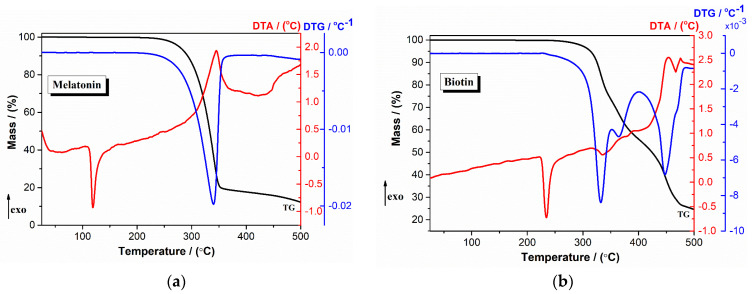

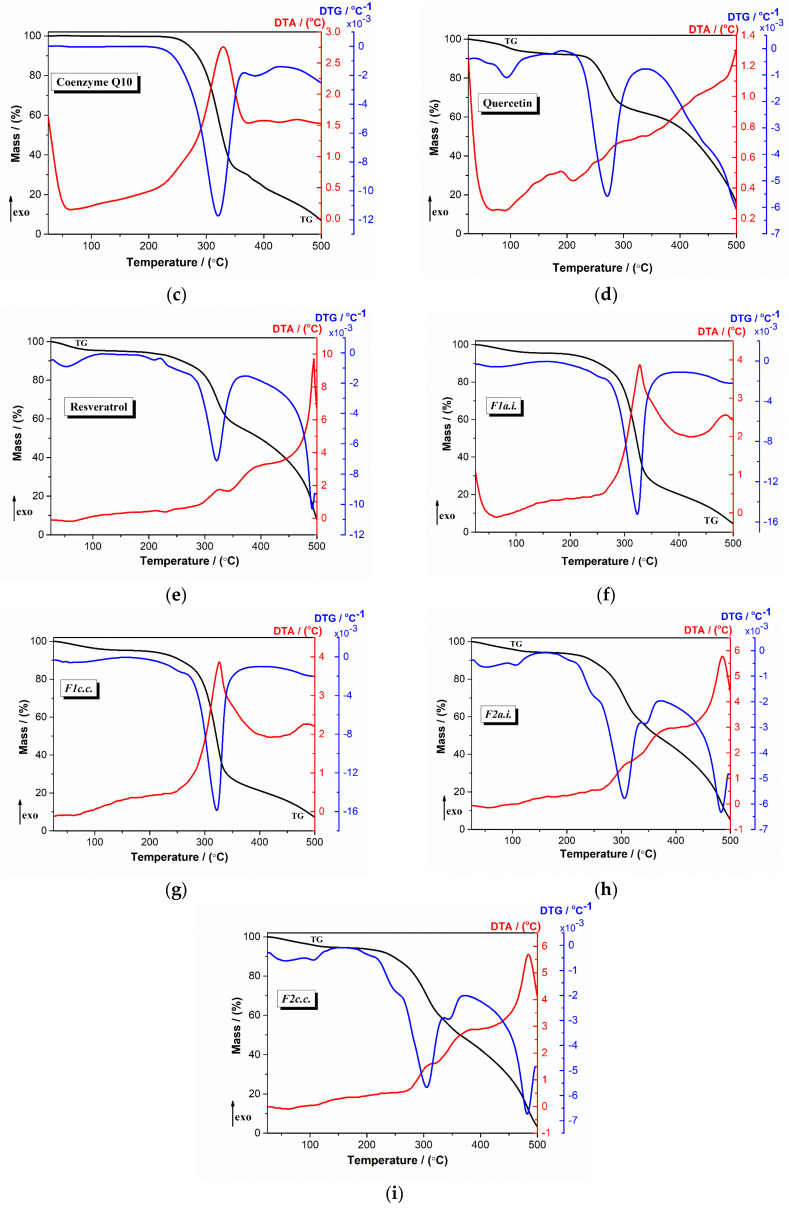
TG/DTG behavior of active ingredients and capsule formulations in an air atmosphere at a heating rate of 10 °C/min (**a**) melatonin; (**b**) biotin; (**c**) coenzyme Q10; (**d**) quercetin; (**e**) resveratrol; (**f**) *F1a.i.*; (**g**) *F1c.c.*; (**h**) *F2a.i.*; (**i**) *F2c.c.*.

TG/DTG analysis of the compounds is represented in Figure 4.

The TG/DTG curve of melatonin (Figure 4a) showed a sharp endothermic transition at 118.7 °C corresponding to its melting point [42]. Subsequently, between 200 and 400 °C, the decomposition process of melatonin occurs (*T_DTG_* = 340 °C, *T_DTA_* = 345 °C). At 234 °C, the biotin compound reached its melting point followed by a significant mass loss between 250 and 500 °C caused by the decomposition process (Figure 4b) [43]. Under a linear heating rate, coenzyme Q10 is stable until 230 °C, when the decomposition process takes place (Figure 4c). The first endothermal peak observed in the TG curve at 95 °C for quercetin (Figure 4d) corresponds to the mass loss due to the elimination of water molecules. The mass loss process observed between 220 and 500 °C indicated the sample decomposition [44,45]. For resveratrol (Figure 4e), the decomposition process overlapped with the melting process, with a maximum peak at 325 °C on the DTA curve.

The TG/DTG curve profiles of capsule formulations (without and with excipients) are relatively similar, with some differences in peak values and the onset temperature decomposition (Figure 4f–i). For both capsule formulations (*F1a.i*. and *F2a.i*.), the disappearance of melting points of individual active ingredients was observed. The results of the TG/DTG analysis of the capsule formulations showed that no new thermal events were observed and, thus, no incompatibility between the constituents existed. The excipients did not modify the thermal behavior of the formulations with active ingredients.

#### 2.1.4. SEM Analysis

**Figure 5:**
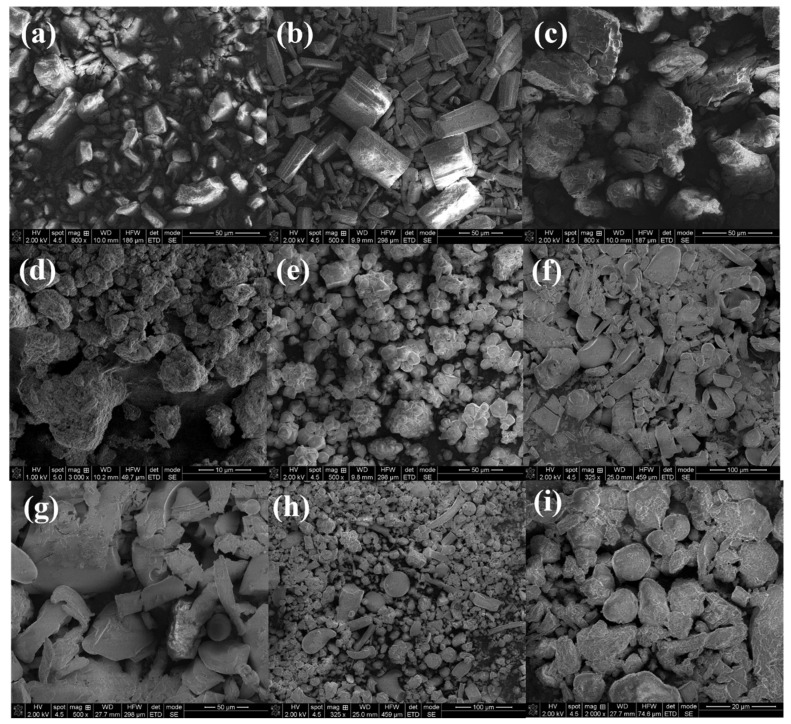
SEM micrographs: (**a**) melatonin; (**b**) biotin; (**c**) coenzyme Q10; (**d**) quercetin; (**e**) resveratrol; (**f**) *F1a.i*.; (**g**) *F1c.c*.; (**h**) *F2a.i*.; (**i**) *F2c.c.*, at different magnifications between 325× and 2000×.

From SEM images, melatonin (Figure 5a), biotin (Figure 5b), and coenzyme Q10 (Figure 5c) were observed as needle-like crystals that formed aggregates [46]. The SEM analysis revealed that quercetin (Figure 5d) and resveratrol (Figure 5e) are individual near-spherical-shaped particles that formed aggregates [47]. SEM images of *F1a.i.* and *F2a.i*. (Figure 5f,h) showed formulations with active ingredients, which are presented as needle strip-like structures and granule aggregates.

### 2.2. Preformulation and Formulation Studies of the Capsules

**Figure 6:**
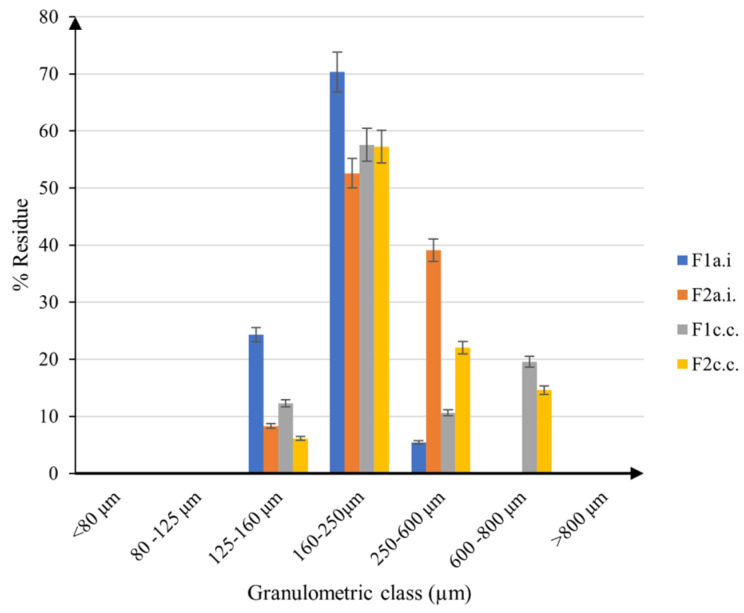
The particle size distribution of the samples.

The particle size distribution for the active ingredient mixtures and capsule content materials are presented in the histogram in Figure 6.

In the mixtures containing only the active ingredients (*F1_a.i_*_._ and *F2_a.i_*_._), most of the particle sizes are below 600 µm, with obvious differences between formulations. *F1_a.i_*_._ proved to have most of the particles in the 125–250 µm interval, while *F2_a.i_*_._ was larger with dimensions that fall within the 160–600 µm range, likely due to the inclusion of resveratrol and quercetin in the formulation. The particle sizes turned out to increase after the capsule excipients were added to the composition, so in the capsule content materials, almost half of the particles have sizes above 250 µm, up to 800 µm. The filling capsule powders present a larger distribution of the particle dimensions, with no significant differences between the two batches, which is not a surprising occurrence as they include the same excipients.

The pharmacotechnical properties of the powders are shown in Table 1.

Concerning the flowability, both *F1_a.i_*_._ and *F2_a.i_*_._ displayed weak flowing behavior, with a flow rate of 2.336 g/s for *F1_a.i_*_._ and 2.118 g/s for *F2_a.i_*_._, typical for active ingredients’ mixtures. Considering that the powder did not flow through the 10 mm nozzle, not even when stirred at 25 rpm, the two powders’ flowability cannot be considered suitable for uniform filling of the capsules. As the particle size distribution also proved, resveratrol and quercetin seem to decrease the flowing performances of the powders, as they are included in *F2_a.i_*_._ in a meaningful quantity. Meanwhile, *F1_a.i_*_._ contains melatonin, which is not found in *F2_a.i_*_._, but its concentration is too low to be able to exert a significant influence on flowability. On the other hand, after the excipients were added, an obvious increase in the flow rate was registered, with values of 3.401 g/s for *F1c.c.* and 3.267 g/s for *F2c.c.* According to Eur. Pharm. [48], an angle of repose ranging from 25 to 30 degrees corresponds to an excellent flow, but since the flow was reported only when using the 15 mm nozzle, great flowability cannot be declared. Still, the compound powders display suitable dynamic properties for the capsules’ filling process, and this is due to the accurate selection of the excipients for both formulations. Even so, *F2c.c.*, based on resveratrol and quercetin, exhibits a reduced flowability in comparison to *F1c.c.*, a predictable behavior considering the data obtained on the initial powders.

The values of CI and HR registered for *F1_a.i_*_._ and *F2_a.i_*_._ are typical for weak-flowing powders in terms of Eur. Pharm. [48] specifications. A CI of 23–35% and an HR above 1.4 are characteristic of very cohesive powders, not suitable for filling hard capsules. The bulk densities of *F1_a.i_*_._ and *F2_a.i_*_._ are extremely similar, but a considerable difference between their tapped densities is observed, proving the contrasting volumetric properties of the samples. This indicates the important influence of resveratrol and quercetin in the composition, in the way that their inclusion decreases the flowability and increases the compactness over a convenient limit. It is also interesting that even though the initial bulk densities are the same and the types and amounts of the added excipient are unchanged, the bulk densities of the compound powders are surprisingly different. The excipients reduced the bulk density of *F1_a.i_*_._ but increased that of *F2_a.i_*_._, demonstrating that some physical interactions between the particles occurred. At the same time, a significant decrease in the tapped densities of both powders was achieved after mixing them with the excipients, highly improving the mechanical properties of the powders. Both CI and HR, calculated for *F1_c.c_*_._ and *F2_c.c_*_._, expressed moderate flowability and compressibility, making them suitable for uniform filling of the capsules.

The moisture content of both *F1_a.i_*_._ (3.95%) and *F2_a.i_*_._ (5.67%) was diminished after the excipients were added, but F2 formulations include a considerably higher amount of humidity in comparison to F1 formulations. Flowability directly depends on the amount and distribution of water in the powder, and increased moisture content causes the powder to aggregate, which results in inconsistent capsule filling.

The adequate selection of the excipient types and amounts was demonstrated by the pharmacotechnical analysis performed on the powders, and suitable mechanical properties of the materials for filling the capsules were achieved after mixing the active ingredients with the excipients.

### 2.3. Capsules Content Antioxidant Activity

#### 2.3.1. DPPH Radical Scavenging Activity

**Figure 7:**
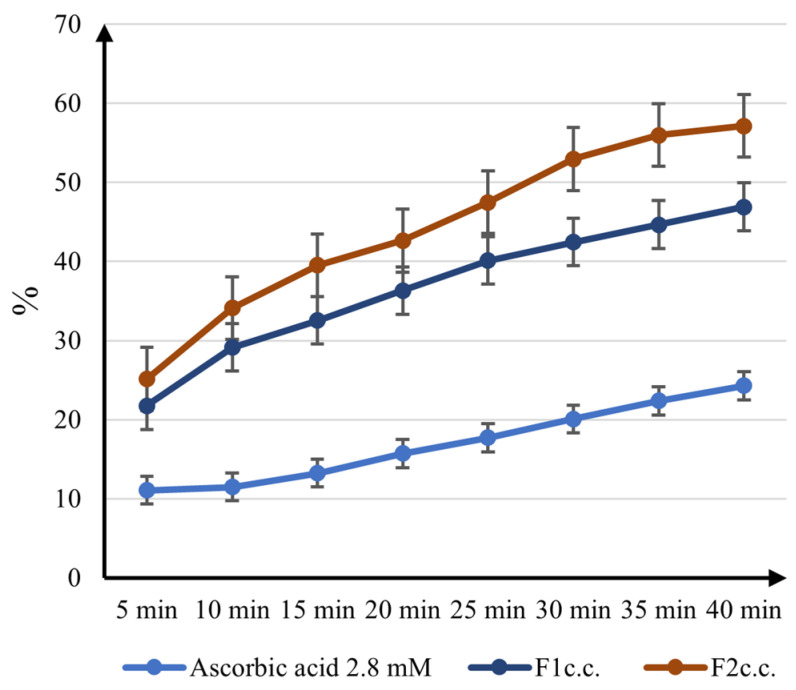
The DPPH radical inhibition of the formulations.

Figure 7 presents the percentage of DPPH radical scavenging activities displayed by both samples and vitamin C 2.8 mM, which was employed as a positive control.

Both formulations proved to be more effective than the ascorbic acid reference, which is estimated to have strong antioxidative activity. *F2_c.c_*_._ exhibits the highest DPPH radical scavenging activity (up to 57.12% at 40 min) due to resveratrol and quercetin content. The results are not necessarily surprising, as both ingredients are well known for their antioxidant abilities. Still, even *F1_c.c_*_._ manifests two times better efficacy (46.88% at 40 min) than vitamin C (24.27% at 40 min), revealing the antioxidant performance of coenzyme Q10. The difference between the two formulations is not considerable, but *F2_c.c_*_._ has a superior electron-donating capacity, confirming the strong antioxidant activity.

#### 2.3.2. ABTS Radical Scavenging Activity

**Figure 8:**
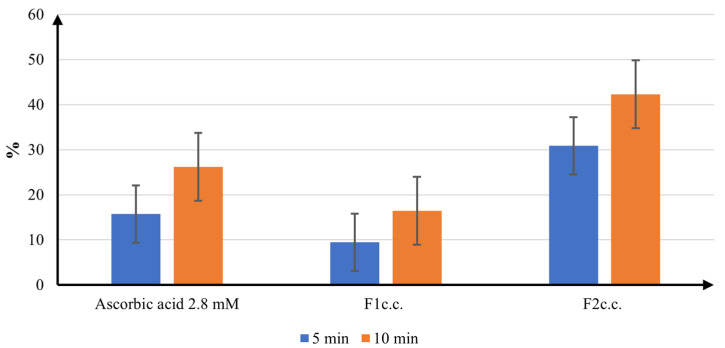
The ABTS radical scavenging activity.

The ABTS radical scavenging activity of the samples is shown in Figure 8.

The ABTS radical scavenging activity revealed a different behavior of the samples, in comparison to the results obtained for DPPH scavenging effect. Nevertheless, *F2_c.c_*_._ manifests the strongest antioxidant efficacy, with a 30.84% inhibition at 5 min and 42.25% at 10 min. On the other hand, the results prove that *F1_c.c_*_._ has a weaker electron-donating ability than the ascorbic acid 2.8 mM, with an ABTS scavenging activity of only 9.45% at 5 min, and 16.43% at 10 min. Due to the quick reaction kinetics and stronger reactivity to antioxidants, it has been shown that the ABTS assay is more sensitive in detecting the antioxidant capacity of the samples. In order to draw an accurate conclusion regarding the antioxidant performance of the studied formulations, a FRAP assay was also determined.

#### 2.3.3. FRAP Assay

**Figure 9:**
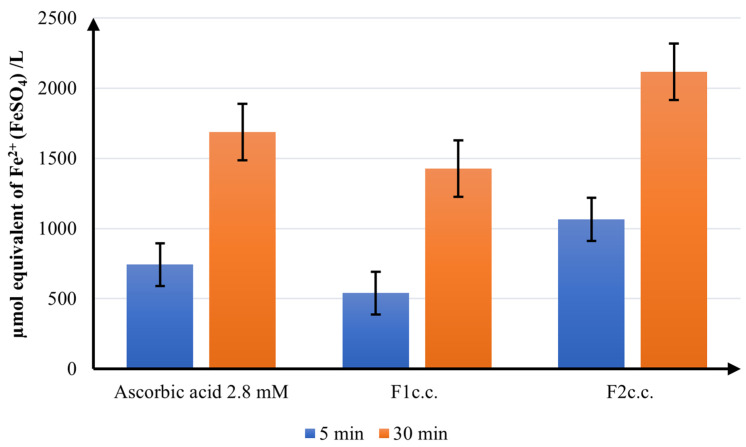
FRAP assay results.

Figure 9 exhibits the FRAP values (µmol equivalent of Fe^2+^ (FeSO_4_)/L) registered at 5 and 30 min for the studied samples.

The samples’ antioxidant performance registered by FRAP analysis confirms the conclusions of the ABTS study, revealing that *F2_c.c_*_._ has the highest antioxidant efficiency. Meanwhile, *F1_c.c_*_._ has the weakest reducing activity, lower than the standard acid ascorbic 2.8 mM used as a reference.

#### 2.3.4. Capsules Quality Control

The obtained capsules’ quality properties were evaluated by mass uniformity and in vitro disintegration time, and the results are shown in Table 2.

Both batches display a suitable mass uniformity in accordance with the imposed regulations, with a slight difference between the formulations (480.31 mg for *F2_c.c_*_._ and 483.17 mg for *F1_c.c_*_._). However, the in vitro disintegration time turned out to vary considerably among them. *F2_c.c_*_._ disintegrates in the aqueous medium in 672 s, a reasonable time for the oral capsules, but *F1_c.c_*_._ disintegrated much faster (458 s) manifesting a better performance, which will lead to superior in vivo behavior [49,50,51,52,53].

## 3. Discussion

As proven by SEM analyses, resveratrol and quercetin tend to gather in aggregates inducing higher sizes of the particles, while the aciculate crystals of melatonin, biotin, and coenzyme Q10 can easily break during the stirring process and, due to their shape, can also readily pass through the meshes of the sieve. The narrow particle size distribution in the small particle range between 125 and 250 µm registered for *F1_a.i_*_._ led to low flowability, which highly increased when the excipients were added. This was manifested due to the broader particle-size distribution found for *F1_c.c_*_._, as the larger particles flow better than the smaller ones, which tend to move slowly [54]. Moreover, in comparison with smaller particles, larger ones have a greater tendency to deform, creating more bonding points during capsule filling. It is anticipated that such plastic and elastic deformation of the bigger particles will increase the surface area of the materials, which is also required for enhanced particle–particle interactions and bonding, and consequently, suitable compaction of the system [55]. The results are in accordance with the findings of Podczeck F and Sharma M [56] who examined the impact of a second ingredient and the size of microcrystalline cellulose particles on the densification of a binary mixture. The compressibility of fine grades of microcrystalline cellulose steadily declines with the addition of spherical (Elcema) and acicular (acetylsalicylic acid) particles. In the case of moderate and large particles, high compactness was detected, but when needle-shaped and spherical particles are added, it drastically declines. Hardy J et al. [57] affirmed that the filling structure of capsule bodies is influenced by the primary particle shape, and combining fine and coarse particles will result in the most favorable packing density.

The cohesive characteristic of initial active ingredient mixtures, observed in their low flowability, was highly improved by the filler and lubricant excipients, and even by the superdisintegrant one, as sodium starch glycolate is already known for its excellent flowing properties [58]. Microcrystalline cellulose was frequently reported to improve the flowing performance of the powders [59,60,61,62], and this is also enforced by the current study. In the case of capsule filling, the optimization of the active ingredient flowability is essential for the development of proper and uniform capsules. Osorio JG et al. [63] demonstrated that the most important elements influencing the weight fluctuation in the filled capsules are the powder flow characteristics. Moreover, Kurihara K and Ichikawa I [64] studied the relationship between filling weight and the powder’s flowing behavior during the filling process. In the present study, *F1_a.i_*_._ and *F2_a.i_*_._ exhibited deficient flowability, which was hard to adjust considering the large amounts of active ingredients in formulations, making the use of high quantities of excipients impossible as bigger capsules are not well accepted by patients. Solving this inconvenience by only using low amounts of a few excipients was difficult and made the selection of the excipients the critical step in the formulation.

The volumetric characteristics of the powders are considerably influenced by the active ingredients, but a significant adjustment was noticed after the excipients were added. Chaerunisaa AY et al. [65] stated that microcrystalline cellulose is characterized by a relatively low bulk density, due to its porous structure, this being the reason for which *F1_c.c_*_._ showed a lower bulk density than *F1_a.i_*_._ Hlinak AJ et al. [66] established that the quantity and type of particular ingredients inside a dosage form determine how a material’s behavior is affected, and according to this, the difference in the bulk densities of *F1_c.c_*_._ and *F2_c.c_*_._ can be explained.

The detected moisture content of the powders clarifies the differences in flowability and compressibility between the two formulations. As *F2_a.i_*_._ possesses a high amount of moisture, likely found in resveratrol or quercetin ingredients, its pharmacotechnical performance was weaker, and even if it was rectified in *F2_c.c_*_._ by the used excipients, it does not reach the characteristic of F1 formulations. Moreover, *F1_a.i_*_._ humidity content was improved by the excipients, thus pointing out the importance and the influence of the excipients in the capsule-filling materials. *F1_c.c_*_._ presents a suitable moisture content for this purpose, while *F2_c.c_*_._ reaches the maximum limit of acceptance concerning the humidity. The results are in agreement with Nokhodchi A’s [67] statement, that higher moisture content causes the bonding strength to decline more quickly than the bonding area can expand, which lowers compressibility and flowing ability. The volumetric properties, flowability, compactness, and particle size distribution of the powders all have a linear relationship and are typical of smooth fluid powders [68].

All three analyses performed to establish the antioxidant character of the formulations certified that *F2_c.c_*_._ possesses the strongest antioxidant capacity, which increases over time, confirming the suitable formulation and accurate selection of the types and amounts of active ingredients, as well as the auxiliary substances. Concurrently, according to the DPPH radical scavenging activity investigation, *F1_c.c_*_._ was found to also exhibit the appropriate antioxidant ability, but it was not supported by the results attained in the other two studies using ABTS and FRAP assays. The contradictory data obtained need to be discussed in relation to other findings mentioned in different research studies. Bibi S et al. [69] affirmed that there is currently no comprehensive and ideal procedure for determining antioxidant performance and obtaining a truthful evaluation of the samples’ antioxidant activity; however, a number of assays (at least three) must be combined. Benzie IF et al. [70] described the FRAP assay as being highly reproducible and sensitive, but at the same time, non-specific. Carocho M et al. [71] stated that the ABTS investigation offers reproductive results, while Ancerewicz J et al. [72] recognized that it is not a suitable model for highly reactive radicals. Kedare, SB et al. [73] favors the DPPH for its efficient reactivity, even with weak antioxidants, but recognizes that DPPH is predisposed to interact with other radicals that exist in the samples. Schaich KM et al. [74] criticize the DPPH method because it is not sensitive to the chemical reaction between the regents. Moreover, Xie L et al. [75] mentioned that DPPH reagent-mediated antioxidant reactions are complex, and reaction curves exhibit a variety of reactivity patterns. Ozcelik B et al. [76] enforce the idea that DPPH reactions are very sensitive to many factors and are not able to offer discriminatory results. Considering the specified information, it is not appropriate to consider that *F1_c.c_*_._ displays a precise antioxidant ability, but further studies are needed in order to establish its real antioxidant performance.

Regarding the evidence of the antioxidant activity of the selected active ingredients, many studies were carried out. Due to its amphiphilic character, which enables it to pass through physiological barriers, melatonin has been identified as an important antioxidant that reduces oxidative damage in both hydrophilic and lipophilic media [77]. In addition to its popular role in treating insomnia or headaches [78,79,80], melatonin is proving to be very useful in preventing illnesses linked to oxidative disturbance, such as cancer [81] and neurological diseases [82,83,84]. In numerous clinical trials, it is being extensively researched for a wide range of different disorders [85]. Johns JR and Platts JA [86] developed an interesting study on the antioxidant ability of melatonin and its metabolites using a donor–acceptor map and managed to prove that melatonin is a powerful biological antioxidant and radical scavenger. Their method unequivocally demonstrates that melatonin has similar power to vitamin E and falls into the good electron donor and bad electron acceptor category. It was noted that the first melatonin-neutral radical metabolite is an even stronger donor than the parent molecule, which has significant ramifications for the biology of the flow mechanism through which melatonin scavenges ROS. A 4-nitro derivative is far outside the range where significant antioxidant capacity should be anticipated; however, other metabolites and the majority of synthetic derivatives still fall within this range. The results of a QSAR study show that the capacity of melatonin derivatives to prevent lipid peroxidation of brain homogenate is significantly associated with their lipophilicity, but only weakly with other molecular characteristics related to donor/acceptor ability. In contrast, these molecular attributes have a substantial correlation with the aqueous phase. As a result, the study somehow justified that the detection of antioxidant properties of melatonin is dependent on many factors that can highly influence the results.

Presently, due to its biological actions revealed by much clinical research, biotin is regarded as a promising antioxidant candidate [87]. Sghaier R et al. [88] disclosed that the cytotoxicity caused by 7-OHC is decreased by biotin, and antioxidant activities return to normal. Protein and lipid oxidation products, as well as excessive ROS generation, were reduced. Biotin lowers the levels of mitochondrial O_2_^•−^ overproduction and normalizes cardiolipin and organic acid levels to partially restore mitochondrial functioning. Additionally, biotin normalizes the synthesis of fatty acids and cholesterol and inhibits apoptosis and autophagy (oxiapoptophagy). Recently, much attention has been oriented toward establishing the recommended dosage for biotin intake, and it seems that many researchers tend to favor higher doses of consumption in order to achieve significant antioxidant activity for biotin [89]. Still, we decided to maintain the biotin concentration in the daily recommended dose for the studied formulations.

Coenzyme Q10 is considered to have a crucial role in mitochondrial bioenergetics. Later investigations showed its presence in different subcellular fractions and in plasma and thoroughly examined its antioxidant activity. The research that underpins the clinical usage of CoQ10 is built on these two purposes [90]. Nevertheless, the greatest inconvenience regarding Coenzyme Q10 use is its limited bioavailability, which leads to discordance data and no clear dosage formulations [91]. For the present study, Coenzyme Q10 varied between the formulations, with 300 mg in *F1_c.c_*_._ and 100 mg in *F2_c.c_*_._. The results pointed out that the formulation containing a low amount of Coenzyme Q10 exhibits better antioxidant properties, even though the excipients are the same, but this could be due to the other active ingredients associated with the formulations.

By preserving the balance of oxidative processes, quercetin has high antioxidant activity [92]. It is granted as one of the most potent antioxidants in different studies [93], but also, its application is constrained by its poor aqueous solubility, low bioavailability, poor permeability, and instability, which leads to minimal absorption into the body [94].

Resveratrol has been shown to possess antioxidant properties based on its chemical structure, which includes a hydroxyl group on the ring and a conjugated double-bond system. According to different studies [95,96,97], antioxidant activity is diminished when three hydroxyl groups have their hydrogen replaced with CH_3_ or when the hydroxyl group is removed.

It is obvious that the association between Coenzyme Q10, biotin, quercetin, and resveratrol produced a positive effect, proving increased antioxidant behavior with satisfying results in the case of *F2_c.c_*_._. Moreover, it can be stated that the proposed formulation had a beneficial influence on its antioxidant performance due to the selected excipients. Meantime, although *F1_c.c_*_._ contains the same excipients, albeit different active ingredients with well-established antioxidant properties, the antioxidant activity was not as expected, proving to be much lower. The results confirm the crucial role of the formulation in assuring the therapeutical effect in the case of combinations of ingredients with different natures and chemical characteristics. The results found for the capsules’ mass uniformity are in accordance with the predicted data obtained in the pharmacotechnical evaluation of the powders. As *F1_c.c_*_._ proved to possess better flowability and compressibility attributes, it was expected that it will fill better the shells in an organized arrangement. The mass uniformity is majorly dependent on the mechanical properties of the primary particles, but it still could be influenced and improved by using a suitable compression pressure [98] that helped in the case of *F2_c.c_*_._ formulation.

Concerning the disintegration properties, a meaningful difference between the formulations was detected. The acceptable disintegration time displayed by both batches in agreement with European Pharmacopoeia specifications confirms the proper selection of the excipients in the formulations. Still, the amount of sodium starch glycolate used as the superdisintgration agent is the same in both formulations, revealing that it is not the only one responsible for promoting the breakdown of the particles [99]. Furthermore, all other excipients are incorporated in similar amounts, which means that even the active ingredients have a great impact on the disintegration performance. It is certified that the mix found in *F1_c.c._* of melatonin:biotin:coenzyme Q10 in a weight ratio of 1:2:60 provides a better disintegration ability than the *F2_c.c_*. composition, which includes quercetin:resveratrol:biotin coenzyme Q10 in a weight ratio of 10:10:1:10.

As a short conclusion, *F1_c.c._* proved to lead to a better pharmaceutical formulation, possessing excellent pharmacotechnical properties imprinted by both the active ingredients and excipients, but, meanwhile, *F2_c.c._* revealed superior antioxidant activity.

## 4. Materials and Methods

### 4.1. Materials

Melatonin (manufacturer Huanggang Saikang Pharmaceutical Co., Ltd., Huanggang, China), biotin (manufacturer Zheijiang Shengda Bio-Pharm Co., Ltd., Taizhou, China), coenzyme Q10 (manufacturer Changsha Phyto Nutrition Inc., Changsha, China), quercetin (manufacturer Shanghai Zhongxin Yuxiang Chemical Co., Ltd., Shanghai, China), and resveratrol (manufacturer Shanghai Zhongxin Yuxiang Chemical Co., Ltd., China) were purchased from Fagron Hellas, Greece. All substances from the pharmaceutical industry were used as received. Avicel PH 102 was purchased from DuPont™ Nutrition and Health, Newark, DE, USA, EXPLOTAB^®^ from JRS PHARMA GmbH & Co., KG, Rosenberg, Germany, and LIGAMED^®^ MF-2-V from Peter Graven NV, Venlo, The Netherlands. Ascorbic acid, potassium persulfate, DPPH (2,2-Diphenyl-1-picrylhydrazyl), ABTS (2,2′-azino-bis(3-ethylbenzothiazoline-6-sulfonic acid), TPTZ (2,4,6-tri(2-pyridyl)-1,3,5-triazine) and Trolox were purchased from Sigma-Aldrich Chemie GmbH, Taufkirchen, Germany.

### 4.2. Methods

#### 4.2.1. Preformulation Studies of the Capsules

In order to achieve suitable capsule formulations, it is mandatory to establish the pharmacotechnical properties of the active ingredient mixtures. Knowing these characteristics will help the accurate selection of the types and amounts of excipients required. Two mixtures were prepared and analyzed: *F1_a.i_*_._, which contains melatonin:biotin:coenzyme Q10 in a weight ratio of 1:2:60, and *F2_a.i._*, which includes quercetin:resveratrol:biotin:coenzyme Q10 in a weight ratio of 10:10:1:10.

*Particle size distribution (PSD)* was determined by sieving analysis on 45 g of each formulation, using a CISA Sieve Shaker Mod. RP 10, produced by Cisa Cedaceria Industrial, Barcelona, Spain. The samples were placed on sieves that were successively arranged from top to bottom in decreasing-size apertures, starting with the 800 µm sieve, followed by 600 µm sieve, then 250 µm, 160 µm, 125 µm, and 80 µm sieves. The set was shaken for 20 min, and the materials retained on each sieve were gathered and weighed.

Regarding *Flowability*, 50 g samples of each formulation were passed through an orifice with a specified diameter, recording the angle of repose, flow time, and rate. The investigations were performed on an Automated Powder and Granulate Testing System PTG-S3, made by Pharma Test Apparatebau GmbH, Hainburg, Germany.

*Compactness* was evaluated using a Vankel Tap Density Tester, made by Vankel Industries Inc., Cary, NC, USA. Calculations were performed for bulk and tapped densities, the Hausner ratio (HR), and the Carr Index (CI). After carefully introducing 50 g of each powder into the graduated cylinder, the bulk volume was measured. The material was then subjected to a predetermined number of mechanical shocks, and the tapped volume was registered. The ratio between the tapped density and bulk density, or HR, and Equation (1) used to determine CI are used to estimate the powder’s propensity to be compressed [100]:

(1) $$CI\left(\%\right)=100\times \frac{\left(\rho_{tapped}-\rho_{bulk}\right)}{\rho_{tapped}}$$

where *ρ_tapped_* is the tapped density (kg/m^3^) and *ρ_bulk_* is the bulk density (kg/m^3^).

A CI value of less than 10 signifies that the powder has great flowability and compactness, and an HR value lower than 1.25 suggests that the powder is freely flowing [59,101,102].

*Moisture content* was assessed as a loss on drying, using the thermogravimetric technique, with an HR 73 Mettler Toledo halogen humidity analyzer from Mettler-Toledo GmbH, Greifensee, Switzerland [103].

#### 4.2.2. Formulation and Preparation of the Filling Materials for Capsules

Considering the results of the preformulation studies and the maximum weight acceptable for the final capsules, selecting the suitable amounts and types of excipients becomes an essential step in product development. The accurate formulation of the capsules’ content will have significant effects on the product’s manufacturability and quality, which will mark its performance, safety, reproducibility, and efficacy.

As the aim was to obtain capsules that can be easily administered orally, maintaining a convenient moderate mass was also an important factor that was taken into account. The quantity per dosage unit was established according to literature data based on the proven beneficial activity.

The final formulations selected are presented in Table 3.

As the blend of the active ingredients displayed weak flowability, a filler with appropriate rheological properties was needed. For this, the microcrystalline cellulose PH 102 was found to be the optimum option. The sodium starch glycolate’s role is to ensure a fast disintegration of the final product, but additionally, it possesses good flowing attributes. Ligamed^®^ MF-2-V was also chosen for its high specific surface area, which will increase the powder flowability.

All components, except the lubricant, were passed through a 20-mesh, weighed, and blended at 30 rpm speed, for 30 min at room temperature in a CMP 12 Plexiglas cube mixer made by Pharmag GmbH (Kliphausen, Germany). Two minutes before the end of the mixing process, magnesium stearate was added.

#### 4.2.3. Physical and Chemical Characterization

A Nicolet 6700 instrument was used to record Fourier Transform Infrared Spectra (FTIR) for powders in the 400–4000 cm^−1^ domain, in KBr pellets. The spectra were recorded in transmittance mode. The measurements had a sensitivity of 4 cm^−1^.

XRD spectra were recorded using a PANalytical Empyrean diffractometer with a Cu X-ray tube (λ Cu Kα1 = 1.541874 Ǻ), at room temperature. X-ray diffractograms were collected using a 0.02° scan step in a Bragg–Brentano geometry, in the range of 20–80°.

Thermogravimetric (TG/DTG) analysis was performed using a Mettler Toledo TGA/SDTA851^e^ instrument in an air atmosphere with a gas flow of 80 mL min^−1^. Heating rates of 10 °C/min were employed.

The morphologies of compounds were studied using scanning electron microscopy (SEM) secondary electron images with a Quanta 3D field emission microscope, which operates at an accelerating voltage of 2 kV in high vacuum mode.

#### 4.2.4. Capsules Content Properties

The obtained materials were evaluated for their pharmacotechnical characteristics by the same methods described above, establishing their *fineness, humidity content, flowing and compactness abilities*, and also their *antioxidant activity* was assessed.

The literature research presents multiple studies on the antioxidant properties of the ingredients selected in the study, but none of the studies determined them based on the final mixture that also contains the excipients. This is the reason we chose to perform these analyses on the filling materials for the capsules, in order to establish if the antioxidant efficacy is maintained in the proposed combinations. The antioxidant activity was assessed by three methods (ABTS, DPPH, and FRAP), and the results were analyzed in comparison with ascorbic acid used as a positive control.

Absorbance measurements in the antioxidant activity studies were performed on a UV-Vis Nicolet Evolution 300 spectrometer, produced by Thermo Electron Corporation, Altrincham, UK.

##### ABTS Radical Scavenging Activity

The total antioxidant capacity was assessed using a previously described method [104]. The cationic radical ABTS^●+^ was generated via the reaction between ABTS and potassium persulfate for 12 h at room temperature, in the dark. The working reagent had an absorbance of 0.70 ± 0.02 at 734 nm. After adding the sample, the decrease in absorbance was recorded for 1 min at 734 nm and the values were computed against a calibration curve made with Trolox. The results were expressed as mmol Trolox equivalents/mg formulation.

##### DPPH Radical Scavenging Activity

This was performed by the method described by Margina D et al. [105]. The absorbances were measured on the mixtures of samples and the DPPH solution, at 505 nm, for 40 min, every 5 min. The results are displayed as the optical density’s decrease percentage from the initial value, after 40 min.

The DPPH scavenging effect (radical scavenging activity (*RSA*%)) was calculated according to Equation (2):

(2) $$RSA\%=\frac{\left(Abs_{0}-Abs_{p}\right)}{Abs_{0}}\times 100$$

where *Abs*_0_ is the absorbance of DPPH solution (reference) at *t*_0_ (initial optical density); *Abs*_p_ is the absorbance of the samples measured at different periods of times *t* (sample optical density).

##### FRAP Assay

The FRAP assay was determined by the method mentioned by Nair VDP et al. [106]. The absorbances were registered at 593 nm for the samples mixed with the FRAP solution at 5 and 30 min after preparation. The FRAP values (µmol equivalent of Fe^2+^ (FeSO_4_)/L) were calculated using Fe^2+^ calibration curves in methanol.

All measurements were performed in triplicate.

#### 4.2.5. Final Capsules Manufacturing and Quality Control

Hard gelatin capsules “0” size, white colored, were used as shells of the product, as their capacity holds approximately 500 mg of powder. They were filled with content, using a FagronLab™ FG1semi-automatic encapsulation device, produced by Gako Konietzko GmbH, Bamberg, Germany. The final two-piece capsules were subjected to specific quality control.

##### Mass Uniformity

This was evaluated according to European Pharmacopoeia [48] specifications by individually weighing 10 filled capsules of each formulation, and then only the empty shells after removing the content. The mass difference is calculated and expressed as the mean value of the filling mass that contains the active ingredients.

##### In Vitro Disintegration Time

This was performed according to compendial criteria [48] on the Erweka DT 3 apparatus made by Erweka^®^ GmbH, Langen, Germany. The test was conducted in distilled water at 37 ± 0.5 °C, on 6 capsules from each formulation. The time required for total disintegration to occur, with only a few capsule shell pieces and no solid center left on the screen, was registered.

## 5. Conclusions

This study comparatively assessed the physical, chemical, pharmacotechnical, and antioxidant properties of the two newly developed capsule formulations containing melatonin, biotin, quercetin, coenzyme Q10, and resveratrol as active ingredients in different combinations and proportions as potential supplements for the human body. Our data provide new insight into a considerable improvement of pharmaceutical supplement formulations with improved antioxidant properties, considering their potential application as ingredients with superior antioxidant activity. Given that all of the active ingredients used are essentially nontoxic, readily accessible in different formulations, and defined as natural products, their topical action is a promising subject for a complete investigation into future protective agents and antioxidant supplements in humans.

## Figures and Tables

**Table 1:** Pharmacotechnical properties of the powders.

Parameter	F1_a.i._	F2_a.i._	F1c.c.	F2c.c.
Flow time (s) *	21.4 ± 1.76	23.6 ± 3.21	14.7 ± 2.37	15.3 ± 2.04
Angle of repose (θ°) *	40.26 ± 2.69	43.12 ± 3.16	27.85 ± 2.12	28.34 ± 1.97
Flow rate (g/s) *	2.336	2.118	3.401	3.267
Bulk density (g/mL)	0.588 ± 0.26	0.589 ± 0.55	0.551 ± 0.18	0.602 ± 0.25
Tapped density (g/mL)	0.833 ± 0.34	0.884 ± 0.41	0.713 ± 0.22	0.806 ± 0.12
Carr Index (CI) (%)	29.411	33.48	22.72	25.31
Hausner’s ratio (HR)	1.417	1.500	1.29	1.33
Moisture content (%)	3.95 ± 1.12	5.67 ± 2.23	3.04 ± 0.85	5.18 ± 1.93

* nozzle: 15 mm, no stirring.

**Table 2:** Pharmaceutical properties of the capsules.

Tested Parameters *	Formulation Code	
	*F1_c.c_*_._	*F2_c.c_*_._
Mass uniformity (mg)	483.17 ± 3.64	480.31 ± 4.29
In vitro disintegration time (s)	458 ± 3	672 ± 7

* mean ± SD.

**Table 3:** Capsule content composition.

Ingredients	Quantity (mg)/Capsule		Role in Formulation
	*F1c.c.*	*F2c.c.*	
Melatonin	5	-	Active ingredient
Biotin	10	10	Active ingredient
Coenzyme Q10	300	100	Active ingredient
Quercetin	-	100	Active ingredient
Resveratrol	-	100	Active ingredient
Avicel PH 102—Microcrystalline cellulose	155	160	Filler
Explotab^®^—Sodium starch glycolate	10	10	Superdisintegrant
Ligamed^®^ MF-2-V—Magnesium stearate	5	5	Lubricant
Total	485	485	

## Data Availability

Not applicable.
